# Doxycycline Inhibits Cancer Stem Cell-Like Properties *via* PAR1/FAK/PI3K/AKT Pathway in Pancreatic Cancer

**DOI:** 10.3389/fonc.2020.619317

**Published:** 2021-02-11

**Authors:** Huijuan Liu, Honglian Tao, Hongqi Wang, Yuyan Yang, Ru Yang, Xintong Dai, Xiujuan Ding, Haidong Wu, Shuang Chen, Tao Sun

**Affiliations:** ^1^ State Key Laboratory of Medicinal Chemical Biology and College of Pharmacy, Nankai University, Tianjin, China; ^2^ Tianjin Key Laboratory of Early Druggability Evaluation of Innovative Drugs, Tianjin International Joint Academy of Biomedicine, Tianjin, China; ^3^ Tianjin Key Laboratory of Extracorporeal Life Support for Critical Diseases, Tianjin Third Central Hospital, Tianjin, China; ^4^ Department of Gastroenterology and Hepatology, General Hospital, Tianjin Medical University, Tianjin Institute of Digestive Disease, Tianjin, China

**Keywords:** protease activation receptor 1, focal adhesion kinase, doxycycline, epithelial–mesenchymal transformation, pancreatic cancer stem cells

## Abstract

Pancreatic cancer stem cells (CSCs) play an important role in the promotion of invasion and metastasis of pancreatic cancer. Protease activation receptor 1 (PAR1) is closely related to malignant progression of tumors, however, its effects on pancreatic cancer stem cell-like (CSC-like) properties formation have not been reported. In this work, the effects of PAR1 on pancreatic cancer stem cell-like (CSC-like) properties formation were studied. PAR1 overexpression can induce CSC-like properties in Aspc-1 cells, whereas interference of PAR1 in Panc-1 cells showed the contrary results. Data on patients with pancreatic cancer obtained from TCGA showed that high PAR1 expression and focal adhesion kinase (FAK) protein considerably affect the prognosis of patients. Further experiments showed that PAR1 could regulate FAK, PI3K, and AKT phosphorylation and the epithelial–mesenchymal transformation (EMT) in Aspc-1 and Panc-1 cells. Doxycycline, as a PAR1 inhibitor, could effectively inhibit the CSC-like properties of pancreatic cancer cells and the FAK/PI3K/AKT pathway activation. Doxycycline inhibits the growth of pancreatic cancer and enhances the treatment effect of 5-fluorouracil (5-FU) in Panc-1 xenograft mouse model. In conclusion, PAR1 promotes the CSC-like properties and EMT of pancreatic cancer cells *via* the FAK/PI3K/AKT pathway. Doxycycline inhibits the pancreatic cancer through the PAR1/FAK/PI3K/AKT pathway and enhances the therapeutic effect of 5-FU.

## Introduction

Pancreatic cancer is a gastrointestinal disease with high mortality and is usually diagnosed at an advanced stage (1, 2). The prognosis of pancreatic cancer is poor, and its 5-year survival rate is only 9% (3). A small population of cancer stem cells (CSCs) in the tumor has the ability to self-renew and maintain the tumor (4). In 2007, Li et al. were the first to isolate and identify pancreatic CSCs (5). Many pathways, such as the hedgehog (Hh) signaling pathway, were upregulated in pancreatic CSCs (6, 7). Epithelial–mesenchymal transformation (EMT) could result in a CSC-like phenotype (8). EMT activation can possibly generate CSCs and affect cancer cell differentiation and metastasis (9).

Protease-activated receptor 1 (PAR1), also known as thrombin receptor (10), is a G-protein coupled receptor. PAR1 mRNA has a higher expression level in pancreatic cancer cells than in pancreatic tissue (11). PAR1 can mediate the tumor microenvironment remodeling, promoting proliferation ability, angiogenesis ability, and malignant evolution in many kinds of tumor (12–14). However, the effects of PAR1 on the CSC-like properties of pancreatic cancer cells have not been reported.

Focal adhesion kinase (FAK) is a cytoplasmic protein tyrosine kinase that regulates cytoskeleton movement and is essential for cell movement (15). FAK is overexpressed in cancer cells (16) and can be activated by phosphorylation to participate in the transduction of multiple signaling pathways and self-renewal of CSCs (17, 18). PAR1 can regulate the self-phosphorylation of FAK in retinal pigment epithelial cells (19). The effect of PAR1 on FAK pathway has not been reported in pancreatic cancer.

Doxycycline is the third generation of semi-synthetic tetracycline broad-spectrum antibiotics (20). It works by inhibiting bacterial protein synthesis (21). Doxycycline has the anti-tumor effect (22–25). However, its molecular mechanism has not been fully elucidated. In this work, the effect of doxycycline on the CSC-like properties of pancreatic cancer was evaluated.

The effect of PAR1 on the formation of CSC-like properties in pancreatic cancer and the effect of doxycycline on pancreatic cancer were evaluated. PAR1 can promote the CSC-like properties and EMT of pancreatic cancer cells *via* the FAK/PI3K/AKT pathway. Doxycycline inhibits the pancreatic CSC-like properties by targeting PAR1 and enhancing the therapeutic effect of 5-fluorouracil (5-FU).

## Materials and Methods

### Cell Culture

The human pancreatic cancer cell lines Panc-1 and Aspc-1 were purchased from KeyGEN BioTECH, and maintained in media recommended by the vendors. The human pancreatic cancer cell lines Panc-1 and Aspc-1 were maintained in Dulbecco’s modified Eagle’s medium (DMEM) supplemented with 10% fetal bovine serum (FBS). The cells were cultured at 37°C with 5% CO_2_ in a humidified atmosphere.

### Gene Transfection

The PAR1-pCDNA3.1 plasmid and siRNA were used for transfection experiments. For transfection, 2.5 μg of DNA and 75 pmol of siRNA were added to 100 μl of the Opti-MEM medium and mixed with 100 μl of Opti-MEM containing 10 μl of Lipofectamine 2000 for 20 min at room temperature. Before transfection, cells were seeded into a six-well plate and transfected with the abovementioned complex for 48 h.

### Western Blot Analysis

The cells were washed with cold PBS, lysed in lysis buffer for 30 min, and centrifuged for 10 min at 4°C. Protein concentration was measured by a BCA (bicinchoninic acid) protein assay kit. Protein samples were separated using 10% SDS-polyacrylamide gel electrophoresis, and electrotransferred onto polyvinyldiene difluoride (PVDF) membranes. After blocking the cells with BSA, the PVDF membranes were incubated overnight at 4°C with primary antibodies, including PAR1 (affinity, 1:1000), FAK (Affinity, 1:1000), p-FAK (Affinity, 1:1000), vimentin (VIM, Affinity, 1:1000), E-cadherin (E-Cad, Affinity, 1:500), PI3K (Affinity, 1:500), p-PI3K (Affinity, 1:500), AKT (Affinity, 1:500), p-AKT (Affinity, 1:500), and GAPDH (Affinity, 1:4000), and with secondary HRP-conjugated goat-anti-rabbit antibodies (Invitrogen, 1:5000). The proteins were visualized by enhanced chemiluminescence and analyzed using Image J software.

### Flow Cytometry

Panc-1 and Aspc-1 cells were seeded into a six-well plate and treated with doxycycline, PAR1-pCDNA3.1 plasmid, or PAR1-siRNA for 72 h. For flow cytometry, Panc-1 and Aspc-1 cells were digested and washed twice with PBS. After fixing the cells with 70% cold methanol and blocking with 5% BSA, they were incubated with primary antibodies CD133 (Affinity, 1:200). The cells were incubated with green fluorescent secondary antibodies. The green fluorescence was analyzed with FACScan flow cytometer, and the result was analyzed by FlowJo software.

### Cell Viability Detection

The viability of pancreatic cancer cells were assessed by MTT assay. Cells (1×10^4^) were seeded in 96-well plates overnight. The experimental groups were treated with doxycycline and combination drugs with different concentrations, and the negative control group was treated with solvent for 48, 72, and 96 h. Then, MTT was added into cells and incubated for 4 h. The synthesized formazan crystals were dissolved using 100 μl of DMSO, and the absorbance was measured at 570 nm. The IC50 of doxycycline was calculated using GraphPad Prism 7.0.

### Invasion Assay

The transwell plate was used for invasion assay. Panc-1 and Aspc-1 cells were suspended and plated into the upper portion of the matrigel-coated transwell chambers, and the bottom chamber was filled with medium containing 10% FBS. The cells were cultured at 37°C for 48 h. The membranes were fixed using 4% paraformaldehyde and stained with 0.1% crystal violet. Then, the cells on the upper of the membranes were removed gently. Cells that invaded through the membrane were counted under a microscope and compared with different drug concentrations.

### Wound Healing Assay

Panc-1 and Aspc-1 cells were seeded into 24-well plate and grown to 70% to 80% confluency. A wound was scratched across each well. The cells were treated with different concentrations doxycycline diluted in non-FBS medium. The wound distance was photographed at 0, 24, and 48 h under a light microscope (Nikon). Three parallel wells were set for each group.

### Immunofluorescence

Panc-1 and Aspc-1 cells were seeded into 24-well plate, treated with 30 and 60 μM of doxycycline, and cultured for 72 h. The cells were washed with PBS, fixed with 4% paraformaldehyde for 20 min, and permeabilizated with 0.1% Triton X-100 for 15 min. After blocking the cells with 5% BSA for 30 min, they were immunoblotted overnight with primary antibodies, including E-Cad and VIM (1:200) and fluorescent secondary antibodies (1:200). E-Cad was labeled with green fluorescence, and VIM was labeled with red fluorescence. Finally, the nucleus was stained with DAPI. Cells were photographed under a laser scanning confocal microscope (LSCM, Nikon). All experiments were performed in triplicate.

### Cloning Formation Experiment

Panc-1 and Aspc-1 cells were seeded into a six-well plate with a density of approximately 300 cells per well. After 24 h, cells were treated with doxycycline, PAR1-pCDNA3.1 plasmid, or siRNA and cultured for 14 days. The colonies were fixed in 4% paraformaldehyde and stained with 0.1% crystal violet. The colony number was counted and compared in different groups.

### Intracellular Ca^2+^ Mobilization Assay

A single cell suspension of pancreatic cancer cells was prepared, and 1.2×10^4^ cells per well were seeded into a 384 plate. After cell adherence, the medium was aspirated, and 25 μl of DMEM containing different concentrations of doxycycline and 25 μl of Calcium Assay kit Loading were added to each well to ensure that the final concentrations of doxycycline were 0, 5, 10, 30, 60, and 100 μM. Advance thrombin solution was diluted to 2 U/ml with DMEM medium. The experimental groups were mixed with 12.5 μl of thrombin solution, whereas the control group was added with 12.5 μl of DMEM medium. Automatic multi-function microplate reader was used to detect the calcium flux signal.

### Animal Studies

Male BALB/C nude mice (5–6 weeks old) were maintained in animal care facilities without specific pathogens. All the animal studies were conducted in accordance with the National Institutes of Health Animal Use Guidelines and current Chinese Regulations and Standards for the Use of Laboratory Animals. All animal procedures were approved according to the guidelines of the Animal Ethics Committee of Tianjin International Joint Academy of Biotechnology and Medicine. The Panc-1 xenografts of tumors (1×10^6^/ml) suspended in PBS were established by subcutaneous injection into the flank. When the tumor volume reached approximately 50 mm^3^, the experimental mice were given doxycycline and 5-FU by gavage, whereas the model and control mice were given distilled water. The volume of nude mice tumors was monitored every 2 days and calculated using the following equation: tumor volume = ab^2^/2 (a, tumor length; b, tumor width). After 60 days of treatment, all mice were euthanized. Tumors, livers, and lungs were collected and fixed with 10% formalin.

### Immunohistochemical Analysis

The slides of tumors from node mice were used to examine E-Cad, VIM, and CD133 expression by immunochemistry. The slides were deparaffinized and re-hydrated, and the antigen was retrieved with 0.01 M citrate buffer. The slides were placed into a wet box after blocking with serum for 30 min and incubated overnight with primary antibodies, including CD133 (1:100), VIM (1:100), and E-Cad (1:100). The primary antibodies were washed away and added with sensitizer for 20 min. The biotinylated goat-anti-rabbit secondary antibody was added for 30 min. Finally, the slides were stained with DAB and hematoxylin, observed under a microscope, and photographed. Staining intensity was scored as follows: none (0), weak brown (1+), moderate brown (2+), and strong brown (3+). The percentages of the positive cells were divided into five classes on the basis of the percentage of tumor cells stained, namely, 0 for no cells, 1 for 1% to 25%, 2% for 25% to 50%, 3 for 50% to 75%, and 4 for >75%.

### Limiting Dilution Assay

Panc-1 cells were diluted to the following densities: 2 × 10^7^/ml, 2 × 10^6^/ml, and 2 ×10^5^/m. The suspension was injected into the right back of the nude mice at 100 μl per injection. The growth status of the tumors was observed, and the tumorigenesis rate of each group was calculated after 2 months.

### TCGA Data Analysis

The representative images of the immunohistochemical assay were obtained from the Human Protein Atlas (https://www.proteinatlas.org). TCGA samples for transcriptional analysis were obtained from UALCAN database (http://ualcan.path.uab.edu/index.html). The expression information of PAR1 and FAK in TCGA pancreatic cancer samples was downloaded from the Human Protein Atlas for correlation analysis. Differential analysis of PAR1 in TCGA pancreatic cancer samples was performed in DECenter (Sanger Box). Then, the significantly upregulated genes (|logFC|≥1.0) were analyzed by GO and KEGG enrichment through the Metascape website (http://metascape.org/). And the proteins that affected by PAR1 were analyzed by PPI analysis (https://string-db.org/).

### Statistics

Each experiment was repeated thrice. Data were presented as mean ± SD and analyzed by GraphPad prism 7. Multiple comparisons were performed by two-way ANOVA. A difference of *P* < 0.05 was considered as statistically significant.

## Results

### PAR1 Promotes the CSC-like Properties of Pancreatic Cancer Cells

**Figure 1:**
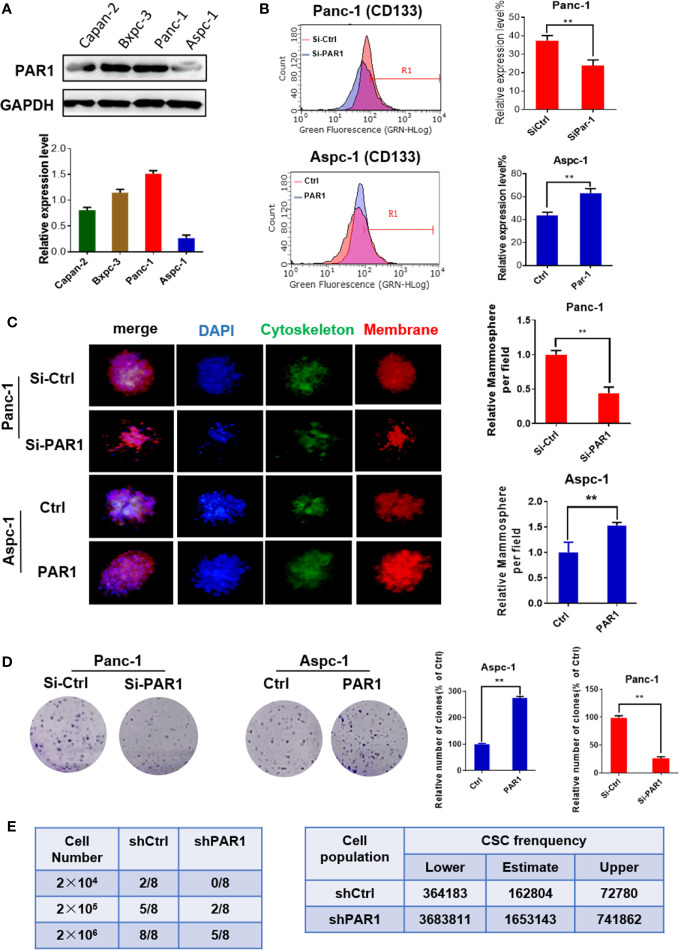
PAR1 promotes the cancer stem cell-like properties of pancreatic cancer cells. **(A)** The expression levels of PAR1 in Panc-1, Bxpc-3, and Aspc-1 cell lines were detected by Western blot analysis. **(B)** Effect of PAR1 on pancreatic cancer stem cell markers CD133 in Panc-1 and Aspc-1 cells. **(C)** Effect of PAR1 on the mammosphere-forming potential of pancreatic cancer cells in PAR1 over-expression and interference experiments. **(D)** Effect of PAR1 on clone formation of Panc-1 and Aspc-1 cells. **(E)** Effect of PAR1 on the tumorigenicity of Panc-1 cells and PAR1 knockdown Panc-1 cells by using limiting dilution assay in node mice (*n* = 8). Cancer stem cell frequency was determined by ELDA (http://bioinf.wehi.edu.au/software/elda). Data are shown as the mean ± SD (**P < 0.01).

The PAR1 expression in pancreatic cancer cell lines was detected by Western blot. The PAR1 expression level was the highest in the Panc-1 cell line but the lowest in the Aspc-1 cell line (**Figure 1A**). Thus, Panc-1 cells were selected for PAR1 knockdown by using siRNA, and Aspc-1 cells were used for PAR1 overexpression by using PAR1-pCDNA3.1 plasmid in the next experiment. The effects of PAR1 on pancreatic CSC marker expression and floating mammosphere-formation ability were detected. CD133 expression level and mammosphere formation ability were reduced in PAR1 interference Panc-1 cells. PAR1 overexpression increases the CD133 expression level and enhances the mammosphere-formation ability in Aspc-1 cells (**Figures 1B, C**). The colony formation experiment indicated that PAR1 overexpression promoted the colony formation of Aspc-1 cells, and PAR1 knockdown inhibited the colony formation of Panc-1 cells (**Figure 1D**). Limiting dilution analysis (using Panc-1 cells and PAR1 knock down Panc-1 cells) showed that pancreatic CSC frequency was significantly reduced in the sh-PAR1 group compared with the control group (**Figure 1E**).

### PAR1 Promotes EMT Progression of Pancreatic Cancer Cells

**Figure 2:**
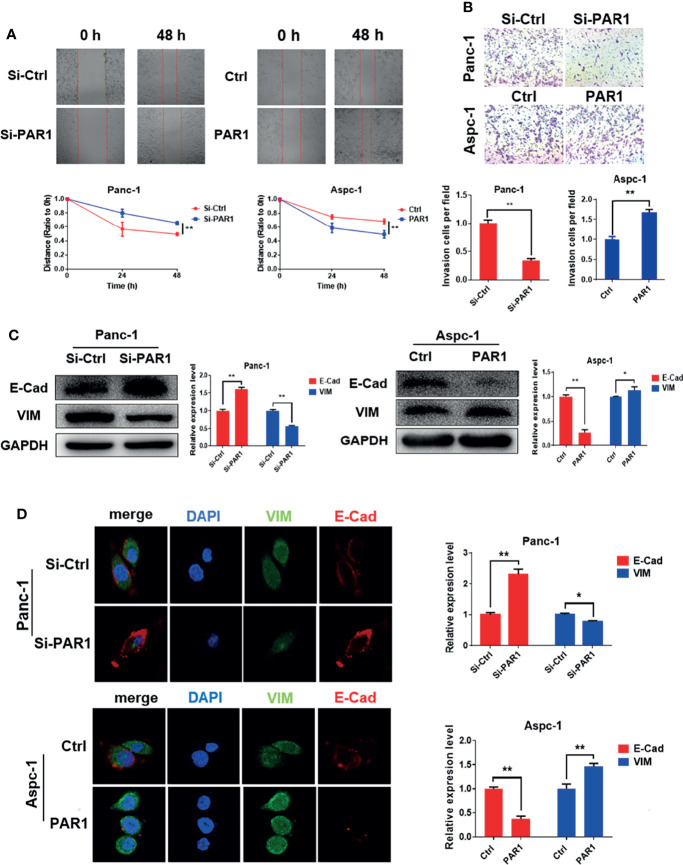
PAR1 promotes EMT progression of pancreatic cancer cells. **(A)** Effect of PAR1 on Panc-1 and Aspc-1 cell migration potential detected using the wound healing assay. **(B)** Effect of PAR1 on pancreatic cancer cell invasion potential by using matrigel-coated transwell assay. **(C)** Effect of PAR1 on the E-Cad and VIM expression detected by Western blot analysis. **(D)** Effect of PAR1 on the E-Cad and VIM expression detected by immunofluorescence. Data are shown as the mean ± SD (*P < 0.05, **P < 0.01).

The stem cell-like features of pancreatic cancer are related to the EMT process of cells. The effect of PAR1 on migration and invasion of pancreatic cancer cells was checked. PAR1 overexpression could promote the migration and invasion ability of Aspc-1 cells (**Figures 2A, B**). PAR1 knockdown could inhibit the migration and invasion ability of Panc-1 cells. Western blot and immunofluorescence experiments showed that PAR1 overexpression could promote the expression of mesenchymal marker vimentin (VIM) and inhibit the expression of epithelial marker E-cadherin (E-cad) (**Figures 2C, D**) in Aspc-1 cells. PAR1 knockdown could inhibit the expression of VIM and increase the expression of E-cad in Panc-1 cells. These results showed that PAR1 could promote the EMT of pancreatic cancer cells.

### PAR1 and FAK Result in the Poor Prognosis of Pancreatic Cancer Patients

**Figure 3:**
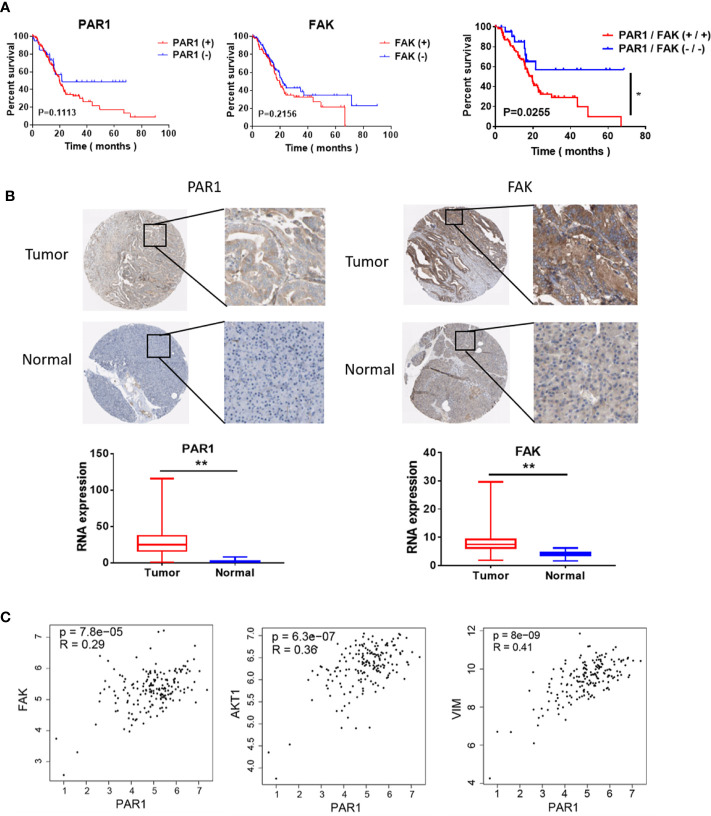
Effect of PAR1 on the pancreatic cancer prognosis. **(A)** Effect of PAR1 and FAK on pancreatic cancer prognosis. Data were obtained from TCGA database. **(B)** PAR1 and FAK expression levels in normal pancreatic tissues and pancreatic cancer tissues. Data were obtained from the Human Protein Atlas online database. **(C)** The correlation analysis between PAR1 and FAK, AKT, and VIM expression of TCGA pancreatic cancer samples from GEPIA (http://gepia.cancer-pku.cn/). Data are shown as the mean ± SD (*P < 0.05, **P < 0.01).

The data of patients with pancreatic cancer were obtained from the cancer genome atlas (TCGA) database ((https://portal.gdc.cancer.gov/) and used to evaluate the effect of PAR1 and FAK in patients with pancreatic cancer. Survival analysis revealed that only PAR1 and FAK double positive expression indicated poor prognosis among pancreatic cancer patients (**Figure 3A**). The immunohistochemical assay from the Human Protein Atlas (http://www.proteinatlas.org/) also showed that the PAR1 (*n* = 176) and FAK (*n* = 176) expression in tumors was higher than that of normal pancreatic tissues (**Figure 3B**). The RNA expression results also showed that PAR1 and FAK expression in tumors was higher than that of normal pancreatic tissues. The expression correlation of PAR1 and FAK, AKT, VIM was analyzed using samples from pancreatic cancer patients from GEPIA (http://gepia.cancer-pku.cn). The expression level of PAR1 and FAK (*P*<0.05, R = 0.29), AKT (*P*<0.05, R = 0.36), and VIM (*P*<0.05, R = 0.41) were positively correlated (**Figure 3C**). Thus, PAR1 may regulate the FAK/PI3K/AKT signaling pathway and result in the poor prognosis of patients with pancreatic cancer.

### PAR1 Could Regulate the FAK/PI3K/AKT Pathway

**Figure 4:**
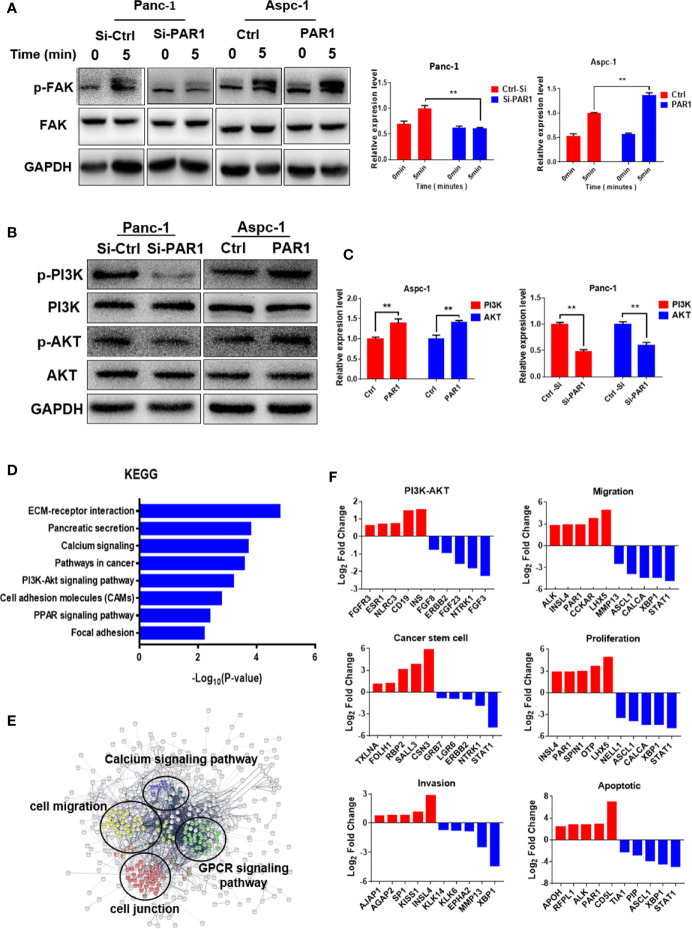
PAR1 can activate the FAK/PI3K/AKT signaling pathways. **(A)** PAR1 activation by thrombin can induce FAK autophosphorylation at Y397 in Panc-1 and Aspc-1 cells. **(B)** Effect of PAR1 on PI3K and AKT phosphorylation in Panc-1 and Aspc-1 cells. **(C)** The statistics results of p-PI3K and p-AKT expression in Panc-1 and Aspc-1 cells. **(D)** The key pathways affected by PAR1 in pancreatic cancer cells. The genomic data for patients with pancreatic cancer were clustered by PAR1 expression level, and the differentially expressed genes were analyzed by GO and KEGG. **(E)** PPI analysis of proteins involved in the pathways affected by PAR1. **(F)** Differentially expressed genes in PI3K/AKT pathway, migration, cancer stem cell, proliferation, invasion, and apoptosis pathways. Data are shown as the mean ± SD (**P < 0.01).

Based on the abovementioned results, PAR1 may regulate FAK phosphorylation in pancreatic cancer. The effect of PAR1 on FAK phosphorylation was detected, and the results showed that PAR1 activation by thrombin could induce the increase in FAK autophosphorylation at Y397 in PAR1-overexpressing Aspc-1 cells (**Figure 4A**). FAK can modulate the PI3K/AKT signaling pathway and promote EMT and CSC formation (26). Thus, the effect of PAR1 on PI3K/AKT phosphorylation was further detected. PAR1 activation by thrombin enhanced PI3K and AKT phosphorylation in PAR1-overexpressing Aspc-1 cells. PAR1 interference could inhibit PI3K and AKT phosphorylation in Panc-1 cells (**Figure 4B**). And the statistics results of phosphorylation PI3K/AKT were shown in **Figure 4C**. After the pancreatic cancer data from TCGA was clustered on the basis of the PAR1 expression level, the differentially expressed genes between the high- and low-expression groups were analyzed. The Kyoto Encyclopedia of Genes and Genomes (KEGG) and Gene Ontology (GO) enrichment analysis results showed that PAR1 affected the ECM-receptor interaction, the calcium signaling pathway, the PI3K/AKT signaling pathway, and focal adhesion in pancreatic cancer cells (**Figure 4D**). PPI analysis result showed that PAR1 affected the function of calcium signaling pathways, cell migration, G-protein coupling receptors, and cell junction (**Figure 4E**). The differential genes involved in cell migration, proliferation, CSCs, and apoptosis were further analyzed (**Figure 4F**). Thus, PAR1 could affect cancer cell invasion, metastasis, proliferation, and CSC formation through FAK/PI3K/AKT pathways in pancreatic cancer.

### Doxycycline Inhibits the CSC-Like Properties of Pancreatic Cancer Cells

**Figure 5:**
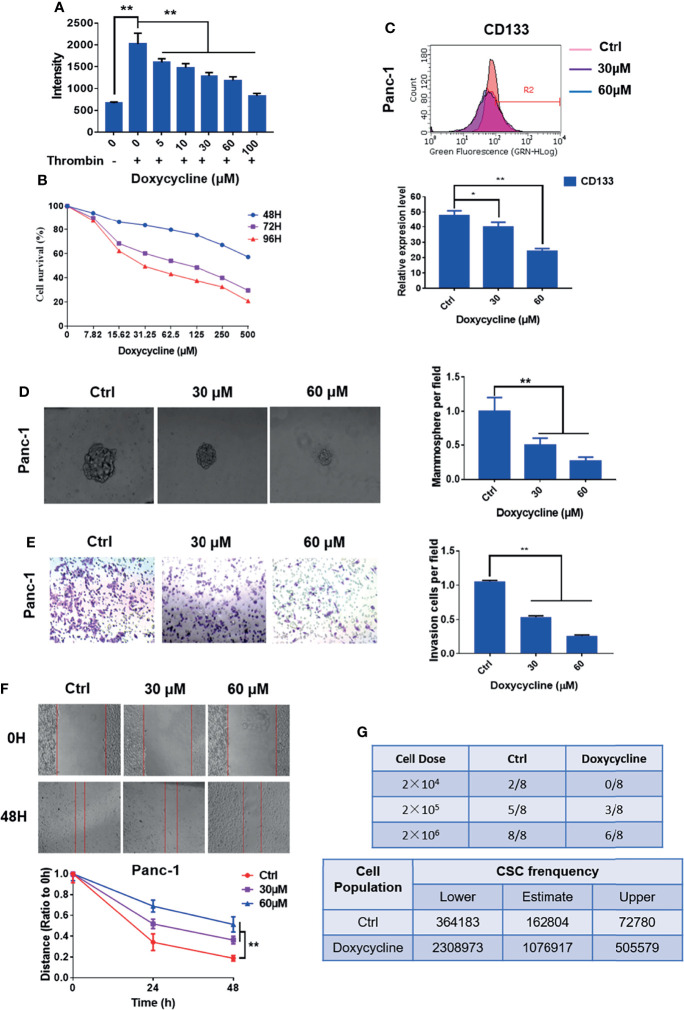
Doxycycline inhibits the cancer stem cell-like properties of pancreatic cancer cells. **(A)** Effect of doxycycline on PAR1 activation stimulated by thrombin in pancreatic cancer cells detected by the Ca^2+^-mobilization assay. **(B)** Effect of doxycycline on pancreatic cancer cell viability after 48, 72, and 96 h treatment. **(C)** Effect of doxycycline on pancreatic cancer stem cell marker CD133 in Panc-1 cells. **(D)** Effect of doxycycline on the mammosphere formation of Panc-1 cells. **(E)** Effect of doxycycline on pancreatic cancer cell migration ability. **(F)** Effect of doxycycline on pancreatic cancer cells invasion ability. **(G)** Limiting dilution assay of pancreatic cancer stem cell from Panc-1 cells after treatment with doxycycline in node mice (*n* = 8). Cancer stem cell frequency was determined by ELDA. Data are shown as the mean ± SD (*P < 0.05, **P < 0.01).

The Ca^2+^ mobilization assay of doxycycline in pancreatic cancer cells showed that doxycycline (Doxy) inhibited intracellular calcium mobilization signals induced by thrombin in a dose-dependent manner (**Figure 5A**). MTT assay results showed that doxycycline inhibited the pancreatic cancer cell activity in a time- and dose-dependent manner (**Figure 5B**). The half inhibition rate (IC50) of doxycycline was 987.5, 99.64, and 50.02 μM after the drug was added for 48, 72, and 96 h, respectively. The effects of doxycycline on pancreatic CSC marker expression and mammosphere formation were examined. The expression levels of CD133 and balloon-formation ability were inhibited by doxycycline in Panc-1 cells (**Figures 5C, D**). The effects of doxycycline on migration and invasion in pancreatic cancer cells were determined. Doxycycline significantly inhibited migration and invasion ability of pancreatic cancer cells (**Figures 5E, F**). In vivo limiting dilution assay also showed that the pancreatic CSC frequency of Panc-1 was significantly reduced in doxycycline-treated group compared with that of the control group (**Figure 5G**). Doxycycline significantly inhibited the CSC-like properties of pancreatic cancer cells.

### Doxycycline Inhibits the FAK/PI3K/AKT Pathway

**Figure 6:**
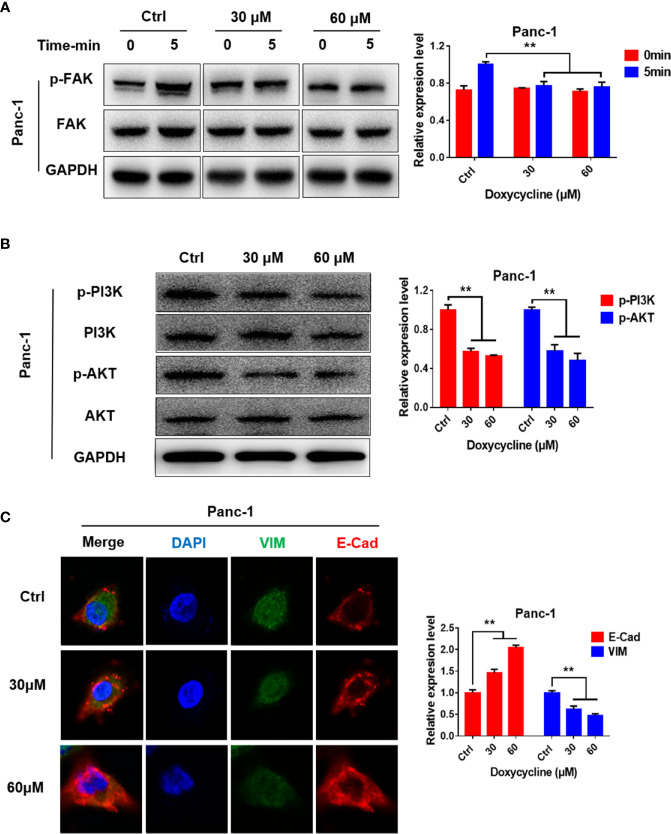
Doxycycline inhibits the FAK/PI3K/AKT signaling pathways. **(A)** Panc-1 cells were treated with doxycycline and stimulated by thrombin for indicated time intervals, and the FAK phosphorylation changes were detected by Western blot analysis. **(B)** Effect of doxycycline on PI3K and AKT phosphorylation in Panc-1 cells detected by Western blot analysis. **(C)** Effect of doxycycline on the E-Cad and VIM expression levels in Panc-1 cells. Data are shown as the mean ± SD (**P < 0.01).

Western blot results showed that the levels of phosphorylated FAK, PI3K, and AKT in Panc-1 cells decreased after treatment with doxycycline (**Figures 6A, B**). Immunofluorescence results showed that doxycycline increased E-Cad expression in Panc-1 cell expression and decreased VIM expression (**Figure 6C**). Thus, doxycycline could inhibit the FAK/PI3K/AKT pathway and EMT of pancreatic cancer cells.

### Doxycycline Inhibits Pancreatic Cancer Growth and Enhances the Therapeutic Effect of 5-FU

**Figure 7:**
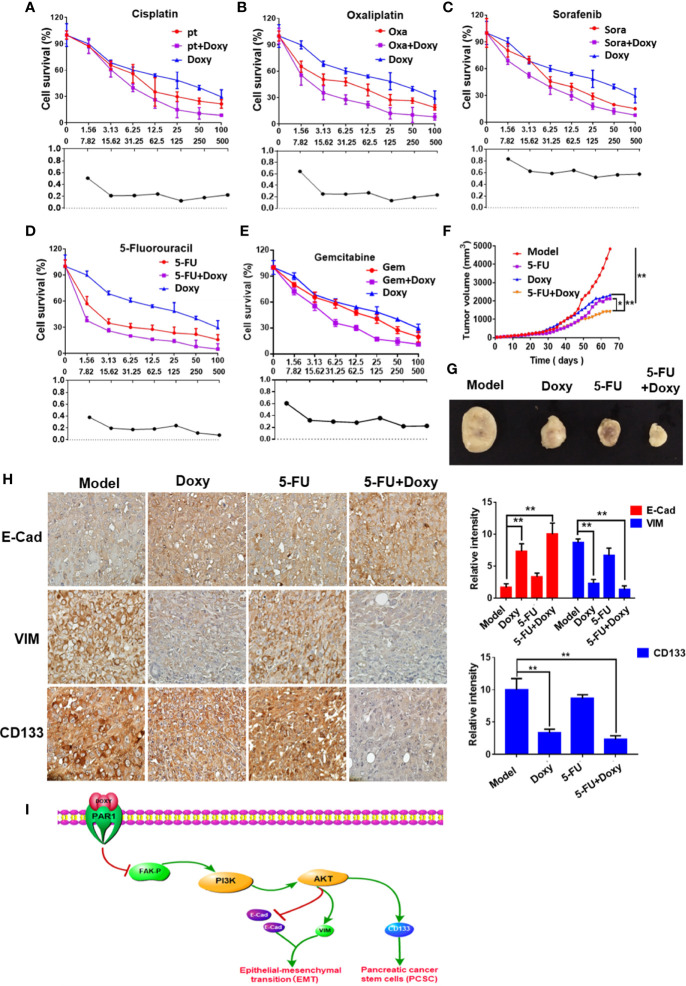
Doxycycline inhibits pancreatic cancer growth and enhances the therapeutic effect of 5-FU. **(A–E)** The sensitization effect of doxycycline for chemotherapeutic drugs in Panc-1 cells after 72 h treatment. **(F, G)** The tumor volume change of Panc-1 subcutaneous xenografts in doxycycline, 5-FU, and the combination treatment group. **(H)** Immunohistochemical staining for E-cad, VIM, and CD133 in tumor tissues. Data are shown as the mean ± SD (*P < 0.05, **P < 0.01). **(I)** Molecular mechanism of doxycycline inhibits cancer stem cell-like properties of pancreatic cancer.

The effects of doxycycline on the viability of Panc-1 cells treated with cisplatin, oxaliplatin, 5-FU, sorafenib, and gemcitabine were detected by MTT assay to determine the sensitization of doxycycline to chemotherapeutic drugs. Treatment with doxycycline effectively enhanced the effect of chemotherapy drugs in comparison with the results obtained when only chemotherapy drugs were used. Doxycycline had synergistic effects with cisplatin, oxaliplatin, 5-FU, sorafenib, and gemcitabine (**Figures 7A–E**). Doxycycline combination with 5-FU showed the best synergistic effect.

Panc-1 subcutaneous xenografts were used to detect the effect of doxycycline and 5-FU to verify the treatment effect of doxycycline and 5-FU on pancreatic cancer *in vivo*. Tumor growth was significantly inhibited in the doxycycline, 5-FU, and the combination treatment group compared with the model group. 5-FU combined with doxycycline had the best inhibitory effect, and the tumor inhibition rate was 80.5% (**Figures 7F, G**). The immunohistochemical assay also revealed that under doxycycline treatment, VIM and CD133 expressions decreased, whereas E-Cad expression increased (**Figure 7H**).

## Discussion

Pancreatic cancer is a disease with high mortality and increasing incidence (27). CSCs can promote cancer invasion, metastasis, and cancer tolerance to chemotherapy (28). The CSC in pancreatic cancer was isolated and identified by Li et al. in 2007. The PAR1 expression level is closely related to the cancer malignant evolution in breast cancer (29). After PAR1 is activated by thrombin, it can mediate cancer cell biological behavior, including the remodeling of tumor microenvironment, and it can promote cancer malignant evolution through downstream molecule and cytokine regulator. The effect of PAR1 on pancreatic cancer cells has not been reported. The effects of PAR1 on the CSC-like properties of pancreatic cancer cells were determined in the present work. PAR1 can promote the expression of pancreatic CSC marker CD133, the microsphere formation ability, and CSC-like characteristics of pancreatic cancer cells. PAR1 can also promote the migration, invasion and EMT of pancreatic cancer cells. Thus, PAR1 can promote the CSC-like properties and EMT of pancreatic cancer cells.

PAR1 can induce FAK phosphorylation in retinal pigment epithelial cells after activation by thrombin, which has not been reported in pancreatic cancer. PI3K is one of the main downstream signaling molecules of the FAK pathway (30). The PI3K/AKT signaling pathway is closely related to cancer cell proliferation, migration, and invasion, and its abnormal activation may result in cancer malignant evolution. FAK may induce EMT (31) and CSC formation through the PI3K/AKT signaling pathway. In the present study, the mechanism underlying the effect of PAR1 on pancreatic CSC formation was investigated by overexpressing and interfering with PAR1 in pancreatic cancer cell lines. FAK, PI3K, and AKT phosphorylation were inhibited after PAR1 interference. PAR1 can activate the FAK/PI3K/AKT signaling pathway in pancreatic cancer cells. It can mediate the occurrence of EMT and the CSC-like properties of pancreatic cancer cells.

In our previous studies, we found that doxycycline is a PAR1 inhibitor. Doxycycline can combine with the PAR1 key amino acid residues of Val257, Leu258, His336, His164, and Leu167, which inhibit PAR1 activity (25). In the present work, we showed that doxycycline can target PAR1 and inhibit the FAK/PI3K/AKT signaling pathway activation in pancreatic cancer cells, EMT, and the CSC-like properties of pancreatic cancer cells. The synergistic effects of doxycycline with chemotherapeutic drugs, such as cisplatin, oxaliplatin, gemcitabine and 5-FU, were evaluated. Doxycycline combined with 5-FU showed the best synergistic effect. Besides, doxycycline and 5-FU can inhibit the growth of the Panc-1 subcutaneous xenografts in nude mice. The immunohistochemical analysis showed a significant increase in E-Cad expression, whereas VIM and CD133 expression levels were significantly decreased. Thus, doxycycline inhibited EMT and the CSC-like properties of pancreatic cancer *in vivo*.

In conclusion, this work showed that PAR1 can promote the CSC-like properties of pancreatic cancer by activating the FAK/PI3K/AKT pathway. As a PAR1 inhibitor, doxycycline can inhibit the CSC-like properties of pancreatic cancer cells targeting the PAR1/FAK/PI3K/AKT pathway and can enhance the therapeutic effect of 5-FU (**Figure 7I**). This work showed that PAR1 may be a therapeutic target of pancreatic cancer and provided insights into pancreatic cancer therapeutic strategies.

## Data Availability Statement

The original contributions presented in the study are included in the article/supplementary materials. Further inquiries can be directed to the corresponding authors.

## Ethics Statement

The animal study was reviewed and approved by the guidelines of the Animal Ethics Committee of the Tianjin International Joint Academy of Biotechnology and Medicine.

## Author Contributions

TS and HL conceived and designed the projects. HL, HT, and HQW wrote the manuscript. HL, HT, HQW, YY, and RY performed the experiments. HL, HT, HQW, XTD, XJD, and SC provided technical and material support. All authors contributed to the article and approved the submitted version.

## Funding

This study was financially supported by the National Natural Science Funds of China (grant nos. 81872374, 81703581, 81972746, 81871972, and 81972629), the Tianjin Science and Technology Project (grant nos. 19JCJQJC63200, 18PTSYJC00060), the Chinese National Major Scientific and Technological Special Project for “Significant New Drugs Development” (grant no. 2018ZX09736-005), and the National Key Research and Development Program of China (grant no. 2018YFA0507203).

## Conflict of Interest

The authors declare that the research was conducted in the absence of any commercial or financial relationships that could be construed as a potential conflict of interest.

## Abbreviations

CSCs, cancer stem cells; Doxy, Doxycycline; PAR1, protease activation receptor 1; FAK, focal adhesion kinase; EMT, epithelial–mesenchymal transformation; 5-FU, 5-fluorouracil; TCGA, the cancer genome atlas; KEGG, tThe Kyoto Encyclopedia of Genes and Genomes; GO, gene ontology; DMEM, Dulbecco’s modified Eagle’s medium; FBS, fetal bovine serum; VIM, vimentin; E-cad, E-cadherin; IC50, the half inhibition rate
