# Corrigendum: Doxycycline Inhibits Cancer Stem Cell-Like Properties *via* PAR1/FAK/PI3K/AKT Pathway in Pancreatic Cancer

**DOI:** 10.3389/fonc.2022.830506

**Published:** 2022-02-24

**Authors:** Huijuan Liu, Honglian Tao, Hongqi Wang, Yuyan Yang, Ru Yang, Xintong Dai, Xiujuan Ding, Haidong Wu, Shuang Chen, Tao Sun

**Affiliations:** ^1^ State Key Laboratory of Medicinal Chemical Biology and College of Pharmacy, Nankai University, Tianjin, China; ^2^ Tianjin Key Laboratory of Early Druggability Evaluation of Innovative Drugs, Tianjin International Joint Academy of Biomedicine, Tianjin, China; ^3^ Tianjin Key Laboratory of Extracorporeal Life Support for Critical Diseases, Tianjin Third Central Hospital, Tianjin, China; ^4^ Department of Gastroenterology and Hepatology, General Hospital, Tianjin Medical University, Tianjin Institute of Digestive Disease, Tianjin, China

**Keywords:** protease activation receptor 1, focal adhesion kinase, doxycycline, epithelial–mesenchymal transformation, pancreatic cancer stem cells

In the original article, there was a mistake in **Figures 2** and **5** as published. The presented figure of siPAR1 (0 h) in **Figure 2A** and the presented figure of Group 30 μM (cells treated with 30 μM of doxycycline) in **Figure 5E** were wrongly presented in the original article. Furthermore, the annotation in the ordinate axis of the statistical figure in **Figures 2B** and **5E** should be “invasion cells per field” not the “passed cells per field.” The corrected **Figures 2** and **5** appear below.

**Figure 2:**
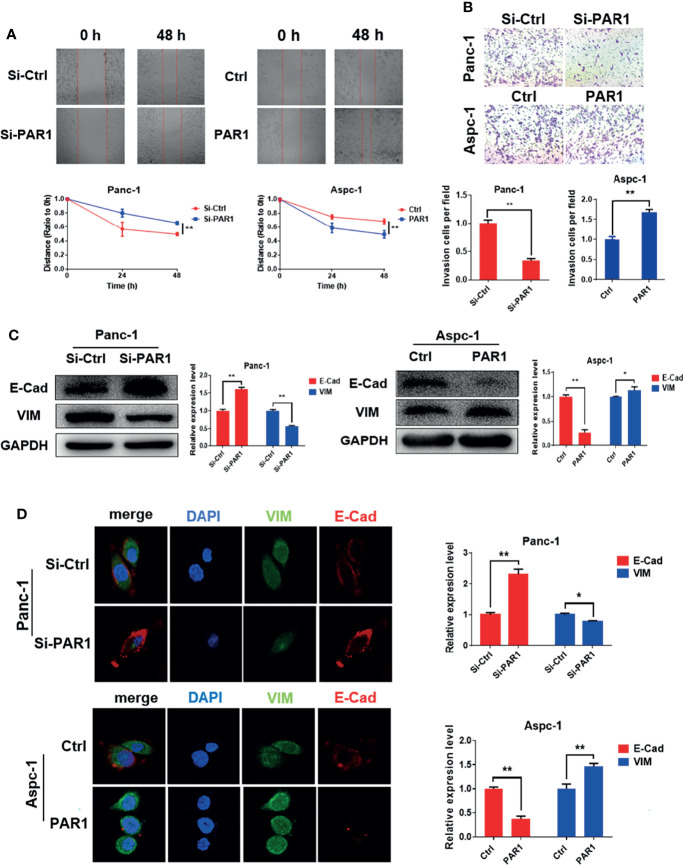
PAR1 promotes EMT progression of pancreatic cancer cells. **(A)** Effect of PAR1 on Panc-1 and Aspc-1 cell migration potential detected using the wound healing assay. **(B)** Effect of PAR1 on pancreatic cancer cell invasion potential by using matrigel coated transwell assay. **(C)** Effect of PAR1 on the E-Cad and VIM expression detected by Western blot analysis. **(D)** Effect of PAR1 on the E-Cad and VIM expression detected by immunofluorescence. Data are shown as the mean ± SD (*P < 0.05, **P < 0.01).

**Figure 5:**
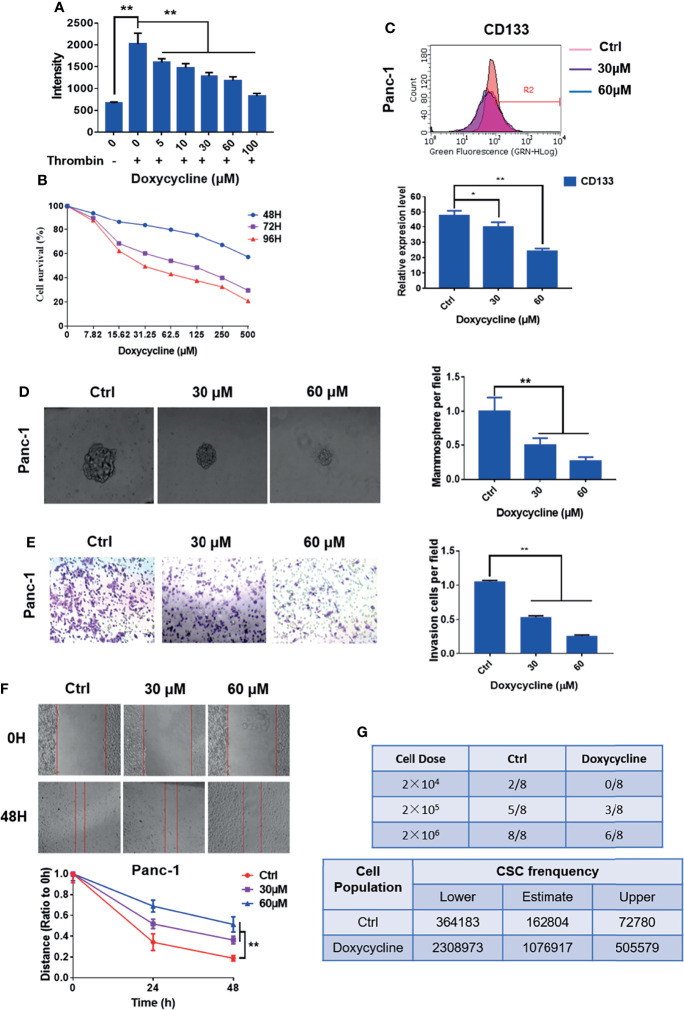
Doxycycline inhibits the cancer stem cell-like properties of pancreatic cancer cells. **(A)** Effect of doxycycline on PAR1 activation stimulated by thrombin in pancreatic cancer cells detected by the Ca2+-mobilization assay. **(B)** Effect of doxycycline on pancreatic cancer cell viability after 48, 72, and 96 h treatment. **(C)** Effect of doxycycline on pancreatic cancer stem cell marker CD133 in Panc-1 cells. **(D)** Effect of doxycycline on the mammosphere formation of Panc-1 cells. **(E)** Effect of doxycycline on pancreatic cancer cell invasion ability. **(F)** Effect of doxycycline on pancreatic cancer cells migration ability. **(G)** Limiting dilution assay of pancreatic cancer stem cell from Panc-1 cells after treatment with doxycycline in node mice (n = 8). Cancer stem cell frequency was determined by ELDA. Data are shown as the mean ± SD (*P < 0.05, **P < 0.01).

The authors apologize for this error and state that this does not change the scientific conclusions of the article in any way. The original article has been updated.

## Publisher’s Note

All claims expressed in this article are solely those of the authors and do not necessarily represent those of their affiliated organizations, or those of the publisher, the editors and the reviewers. Any product that may be evaluated in this article, or claim that may be made by its manufacturer, is not guaranteed or endorsed by the publisher.

